# Deformities of the Hard Palate in Degenerates

**Published:** 1895-12

**Authors:** Frederick Peterson


### International Dental Journal.

Vol. XVI. December, 1895. No. 12.

### Original Communications.[1]

### DEFORMITIES OF THE HARD PALATE IN DEGENERATES.[2]

BY FREDERICK PETERSON, M.D.[3]

#### DEGENERATION.

The subject of degeneracy in the human race has for some years been exciting much interest and discussion among scientific men. I may be pardoned therefore for bringing forward a few general considerations of degeneracy as preliminary to what I have to say with regard to one of the physical signs of that condition which is of especial importance to the body before which this brief paper is read.

Degeneracy may be defined as a marked deviation from the normal original type or standard. We recognize it, as a rule, in its effects upon the intellectual life, in the deviations from the intellectual habits and social conduct which we hold in common with our fellows. To the class of degenerates not only belong

[1] The editor and publishers are not responsible for the views of authors of papers published in this department, nor for any claim to novelty, or otherwise, that may be made by them. No papers will be received for this department that have appeared in any other journal published in the country.

[2] Read before the New York Odontological Society, October 15, 1895.

[3] Neurologist to Randall's Island Hospital for Idiots; Pathologist to the New York Insane Asylums; Chief of Clinic, Nervous Department, Vanderbilt Clinic, College of Physicians and Surgeons, New York.

many criminals, idiots, and insane individuals, but also the great majority of persons whom we call cranks or eccentrics, the people who live among us a sort of original life, with peculiarities of mental habit and conduct, and whom we characterize as feeble-minded, odd, quaint, queer, or singular.

A man of talent or of genius often presents eccentricities of the kind to which we refer, but such deviation from the original normal standard need not be morbid in character; it may be a deviation towards a higher and better standard, recognized by his contemporaries or posterity to be such, and to which we on our parts try in the end to conform. It might be difficult at times to distinguish between the eccentricities of genius and the eccentricities of degeneracy. There are one or two indications or tests which will aid us in this. One of the indications—in fact, the chief test—of a normal state is naturally conformity to the social condition in which a man lives. This test applied by itself, however, does not exclude talented individuals and geniuses. Another criterion must be applied to these cases. Is there conjoined with the eccentricity a morbid self-centring of his interests? It is in individuals who concern themselves little with the affairs of the world, but much with personal and selfish matters, that eccentricity of intellectual habit or conduct warrants a grave diagnosis. Now, one of the essential characteristics of degeneracy is its inclusion of transmissible elements, so that the degenerate individual not only bears in himself the germs which render him more and more incapable of fulfilling his own functions in human life, but by his hereditary bequests he menaces the intellectual stability of his descendants.

So much for the definition of the term degeneracy. We will pass on to a brief consideration of the indications of degeneracy.

### STIGMATA OF DEGENERATION.

The indications of degeneracy are known as stigmata hereditatis, or stigmata of degeneration. They may be defined as anatomical or functional deviations from the normal, which in themselves are usually of little importance as regards the existence of an organism, but are characteristic of a marked or latent neuropathic disposition. Much study has of late years been devoted to these indices by many investigators, particularly in their relation to insanity, idiocy, and criminal anthropology, and it behooves all who have to do with the development and care of the human body in any particular, and this refers especially to men of the medical and allied professions, to familiarize themselves with these signs of degeneration in so far as they concern their own special provinces of work. These stigmata are vices of functional and organic evolution. The deviations from the normal may be in the way of excesses or arrest of development. They must be distinguished from the deficiencies or deformities produced by accidents at birth or by disease. I have said that these stigmata are anatomical and functional. But it is more convenient to divide the functional group into physiological and psychic classes. It is the latter which we are more apt to observe in our social relations with degenerate individuals. The psychic stigmata are always characterized by a want of balance or lack of proportion between certain undeveloped or excessively developed faculties and other faculties which are normal. Defect of moral sense, attention, memory, will, judgment, or excess of musical or mathematical aptitudes, may be cited as instances of psychic stigmata. Hence the three following divisions may be made of all the degenerative indices:

(1) Anatomical Stigmata.
(2) Physiological Stigmata.
(3) Psychical Stigmata.

### ANATOMICAL STIGMATA.

Cranial anomalies.
Facial asymmetry.
Deformities of the palate.
Dental anomalies.
Anomalies of the tongue and lips.
Anomalies of the nose.
Anomalies of the eye.
- Flecks on the iris, strabismus, chromatic asymmetry of the iris.
- Narrow palpebral fissures.
- Albinism.
- Congenital cataracts.
- Pigmentary retinitis.

Anomalies of the ear.
Anomalies of the limbs.
- Polydactyly.
- Syndactyly.
- Ectrodactyly.
- Symelus.
- Phocomelus.
- Excessive length of the arms.

Anomalies of the trunk.
- Hernias.
- Malformation of the breasts and thorax.
- Dwarfishness.
- Giantism.
- Infantilism.
- Femininism.
- Masculinism.
- Spina bifida.

Anomalies of the genital organs.

Anomalies of the skin.
- Polysarcia.
- Hypertrichosis.
- Absence of hair.
- Premature grayness.

### PHYSIOLOGICAL STIGMATA.

Anomalies of motor function.
- Retardation of learning to walk.
- Tics.
- Tremors.
- Epilepsy.
- Nystagmus.

Anomalies of sensory function.
- Deaf-mutism.
- Neuralgia.
- Migraine.
- Hyperæsthesia.
- Anæsthesia.
- Blindness.
- Myopia.
- Hypermetropia.
- Astigmatism.
- Daltonism.
- Hemeralopia.
- Concentric limitation of the visual field.

Anomalies of speech.
- Mutism.
- Defective speech.
- Stammering.
- Stuttering.

Anomalies of genito-urinary function.
  Sexual irritability.
  Impotence.
  Sterility.
  Urinary incontinence.
Anomalies of instinct or appetite.
  Merycism.
  Uncontrollable appetites (food, liquor, drugs).
Diminished resistance against external influences and diseases.
Retardation of puberty.

PSYCHICAL STIGMATA.

Insanity.
Idiocy.
Imbecility.
Feeble-mindedness.
Eccentricity.
Moral delinquency.
Sexual perversion.

I have thus enumerated the stigmata of degeneration in order to show how numerous they are and in what great variety they exist, not only in the physical organism but in the realms of the general functions of the body and the higher functions of the nervous system.

All this is merely an introductory to the particular subject which I have been invited to present to your attention this evening,—viz., Deformities of the Hard Palate in Degenerates. While the palate occupies but a small place in this great category of hereditary stigmata of all kinds, it is one of the anatomical group, and this group is for many reasons the one of greatest importance. In this group, too, it occupies a distinctive place as being among the most striking, frequent, and significant of the anomalies. I will not say of the palate what Dr. Amadée Joux said of the ear, "Show me your ear, and I will tell you who you are, whence you came, and where you go;" but I will say, "Show me your palate, and I will probably be able to tell whether you belong to the great class tainted by heredity, comprising many insane, imbecile, feeble-minded, criminal, eccentric, epileptic, hysterical, or neurasthenic individuals."

Therefore it is to you dentists, who see before you in your daily vocation large numbers of these organs, that the subject now presented to you must have a peculiar interest. Doubtless you are not always brought into contact with the psychic life of the individuals who come to you for treatment; you may not often be consulted as to conditions outside of your own field of work. But at any rate you may frequently do an immense amount of good, when you discover a pathological palate, by calling the attention of a family to an indication of degenerative proclivity in one of its members. If it be a young person, you may be the first to observe a stigma, and the first to direct attention to a peculiarity which may have enormous significance as regards his future bringing up and care. You will recognize the need of examining him for other stigmata,—anatomical, physiological, or psychic,—and the necessity of a special line of education and development if he prove to be feeble-minded or have a tendency to a psychopathic state, and of throwing about him safeguards against the evolution of eccentric, hysterical, neurasthenic, or other neuropathic conditions.

Before describing the pathological palate I would say a few words about the

NORMAL HARD PALATE AND ITS VARIATIONS WITHIN NORMAL LIMITS.

The palatine process of the superior maxilla forms the anterior two-thirds of the hard palate and part of the floor of the nose. The posterior third of the hard palate is completed by the horizontal plate of the palate bone.

On the palatine surface we find the palatine groove for the descending palatine vessels and nerve, numerous foramina for vessels entering the bone, and pits made by small palatine glands.

Immediately behind the middle incisor teeth is the anterior palatine canal in the palatine suture. The palatine suture is a very rough one. In young bones, as in the facial skeleton I show you here, you will observe traces of the premaxillary suture, which separates the part of the maxilla containing the sockets for two incisor teeth. This is the premaxillary or intermaxillary bone, which is, in some of the lower animals, a separate bone.

Ossification of the upper jaw begins about the seventh week of fœtal life, and progresses so rapidly that the number of ossification centres has never been accurately ascertained. Five of these centres are well known, however, and among them are one for the palatine process and one for the premaxillary portion. The palate bone has one centre, from which the ossification spreads into its perpendicular portion and inward along its horizontal plate.

The arch of the hard palate presents considerable variation within strictly normal anatomical limits. A large, wide, moderately high vault is what may be called a normal standard. It means the highest evolution, judging from the fact that the mouth-cavity increases in capacity as we ascend the vertebrate series. Deviations from that standard are not at all infrequent, as you well know, and yet such deviations may be normal. Thus, the palate may be low and broad or it may be high and narrow; it may be short or long in its anterior-posterior diameter; it may be ridged unduly along the palatine sutures, or it may present marked rugosities on its surfaces, especially in the anterior region; yet these variations are normal. Probably we may look upon these peculiarities as a species of compensatory development. Just as in a study of heads we find some very long and low, and others short and round and high, and recognize the fact that shortness in one dimension is compensated for by a corresponding increase in another. So we may regard variations in palatine diameters.

### DEVELOPMENT OF THE NORMAL HARD PALATE.

Nothing is more interesting than a study of the development of the mouth and face. The face is, in a manner, chiefly an evolution of the mouth-cavity. The face is characteristic of the higher vertebrates, and increases in importance the higher we ascend in the scale. In some of the lower orders of animals there is no projecting face, but merely an area on the ventral aspect of the head, with a mouth and nasal pits.

The evolution of the face depends upon four factors,—viz., enlargement and fusion of the oral cavities; partial separation of the oral and nasal cavities; growth and specialization of facial region, the elongation of the jaws being the most conspicuous feature; and the development of the nose. The position of the face and oral cavity is determined originally by the head-bend in the embryo, but as development progresses the oral and facial parts enlarge out of proportion to the rest of the head so as to project in front of the fore brain, and the bulk of the face is great in proportion to that of the cranium (in lower animals). In mammals the brain increases greatly in size until it extends over and above the face, and in man finally covers the facial region.

Now, the mouth-cavity is formed in this wise: At first there is merely an oral depression in the ectoderm, on the other side of which is the endoderm, with its alimentary canal and air-passages. Rupture takes place in this depression after a time, the mouth and pharynx communicate, and a single wide cavity is formed. The tongue develops from the floor of the pharynx.

At first the nasal pits open freely and widely into the mouthcavity. Then a partition grows down to separate the two nasal cavities, and after this the mouth comes to be divided into an upper and lower mouth by the appearance of two shelf-like projections at the sides of the mouth, which, growing towards each other, finally unite as the hard palate. The upper mouth, for respiration, is added to the nasal cavities, while the lower mouth is left for alimentary uses.

The hard palate, then, is formed by these two shelf-like processes from the inner side of the maxillæ, and these grow at first obliquely downward, with the tongue between them. Then the lower jaw begins to grow with great rapidity, and draws down the floor of the mouth and lowers the tongue. The palate shelves assume a horizontal position and meet and unite in the middle line,—first in front and later behind. Union begins at eight weeks in the fœtus, and is completed in the hard palate at nine weeks, in the soft palate at eleven weeks. Soon after this the nasal septum unites with the palate.

I have dwelt upon these facts in the development of the face, mouth, and hard palate in order to make clear the peculiar way in which the hard palate takes its origin, and to show the marked developmental relations between the brain, mouth, face, and the various walls which serve to separate and divide them. It is evident that arrest or error of development in any one of these parts must inevitably affect, in one way or another, the others.

### THE PATHOLOGICAL PALATE.

In passing to the consideration of the pathological palate, I would premise that I am fully aware of the difficulty of making hard and fast rules for the differentiation of the abnormal from the normal, and of the danger of making too positive statements. I have searched literature very carefully, and find that very little, indeed, has been written upon the subject, and it is, in a way, making new paths for others to follow and improve upon to bring this matter to your attention. The phrase Gothic palate is, to be sure, frequently mentioned in literature, but only casually. Several papers have been written upon the torus palatinus. I have examined the hard palate in normal and abnormal states for over eleven years. I presume as many as a thousand insane, one hundred criminals, six hundred idiots, and five hundred or more neuropaths of other kinds would fairly represent the number of pathological conditions from which I have gained some of the expert experience which has led me to present this paper for your consideration, and I mention these facts merely as a justification of my attempt to make a sort of classification of the kinds of pathological palates which should be regarded as stigmata of degeneration. The word Gothic having been so long in use, and the hard palate being much like an arch or roof, I have followed architectural nomenclature in the classification offered:

PATHOLOGICAL PALATES.

(A) Palate with Gothic arch.
(B) Palate with horseshoe arch.
(C) The dome-shaped palate.
(D) The flat roofed palate.
(E) The hip-roofed palate.
(F) The asymmetrical palate.
(G) The torus palatinus.

As illustrations of these varieties of abnormal palate, I present for examination seventeen casts of the hard palate, mostly selected from among the four hundred and fifty idiots on Randall's Island. I am under great obligation to Dr. Walker and his assistant, Dr. Turner, for making the moulds and casts in these cases.

The seven varieties named are to be looked upon as types merely. Each type will be found to present variations and combinations with other forms. Thus, the Gothic arch may have a low or high pitch, and be short or long. The horseshoe arch (a familiar one in Moorish architecture) is always easily distinguished; but owing to its conformation a cast cannot well be taken of it to show it in perfect outline. The dome-shaped palate may be high or low, may be combined with asymmetry or torus. The presence of a torus in the Gothic variety is apt to destroy the purely Gothic form, and may cause it to resemble the flat-roofed palate. Under the heading of flat-roofed palate I should include all such palates as are nearly horizontal in outline (of which I have not a good specimen to exhibit), as well as those with inclined roof sides but flattened gable. In the hip-roofed palates we have the sloping sides as usual, but also a marked pitch of the palate roof in front and behind. Occasionally one meets with a palate of this kind with so remarkable a pitch from before backward that it is almost like a Gothic roof turned about, so that the gable runs transversely. Asymmetry in the palate is commonly observed in many of the previously described forms, but occasionally is the only noteworthy peculiarity. It is usual to find asymmetry of the face and skull in cases with an asymmetrical palate.

The torus palatinus (Latin, *torus*, "swelling") was first mentioned by Chassaignac as a medio-palatine exostosis. It is a projecting ridge or swelling along the palatine suture, sometimes in its whole length, sometimes in only a portion of its course. It is always congenital. It varies considerably in its shape and size, so that as many as five or six different species of torus are recognized. It may be wedge-shaped, narrow, broad, very prominent, or irregular. I have said nothing about cleft-palate, for I am not sure that it may be classed among the well-marked stigmata of degeneration. I have found but two or three cleft-palates among the four hundred and fifty idiots and imbeciles on Randall's Island, while a number of cases of this kind with which I have come in contact in my professional life were very far from being degenerates. However, it would seem that there is great need of a faithful study of a large number of cases of cleft-palate in relation to the question of degeneracy.

### THE ETIOLOGY OF THE PATHOLOGICAL PALATE.

When we come to investigate the causes which lead to the formation of an abnormal palate we meet with much difficulty. Much could be learned, probably, by the examination of large numbers of newly-born children. As far as my own experience goes, the kinds of palate to which I have devoted this paper are always congenital. I am aware that changes are produced in the palate by mouth-breathing, and that some specialists in diseases of the nose and throat are inclined to ascribe most palatal deformities of the character above mentioned to this cause; but the evidence is convincing that another origin, and that a prenatal one, must be assigned to these deformities. We must look rather to modifications occurring during fœtal development, during the evolution of the child, modifications brought about by arrests or errors of development, not so much, perhaps, in the palate itself as in the brain, the base of the skull, and the intricate structures that make up the face and nose, because the relative positions and interdependence of these parts is so marked that any alteration in one must in somewise affect the development and configuration of all the others.

As to the ultimate cause of these modifications which give rise to stigmata of every kind, that must be sought in the nervous mechanism which governs heredity. As the evolution of our bodies as well as our minds depends upon the brain and spinal cord and the countless nerve-filaments which radiate from them to every tissue, so the nervous system plays the most important part in the influences which have to do with heredity. The nervous co-ordinations must be rearranged by strong stimuli in order to reproduce the hereditary impulse. This is why traits acquired by us in our individual lifetimes are not apt to be inherited by our descendants. If a person loses an arm, his children are not deprived of that useful member, for the nervous mechanism of development which has for ages produced arms in their proper places, and which is fixed in the powerful hereditary impulse of the race, has not been changed. So in the breed of dogs whose tails have been cut off for countless generations, not one is born without a tail, because the nervous co-ordinations governing the evolution of the tail bear down with all the hereditary force of the race since its first beginning (when the tail existed, though the animal was legless) to keep it in existence. If in some way we could reach the nervous mechanism which is responsible for the evolution of the tail, we might modify or even prevent its development.

It is therefore some derangement of the nervous mechanism governing heredity which brings about deviations from the normal type which gives rise to these anatomical, physiological, and psychic anomalies which we designate as the stigmata of degeneration.

How is the nervous mechanism of heredity deranged? It may be readily and profoundly deranged in a variety of ways, for instance, by poisons. Thus alcohol disarranges the nervous mechanism of heredity in such a way that the descendants may suffer from drink-craving, from idiocy, insanity, epilepsy, hysteria, neurasthenia, from shattered nervous systems, for at least three generations, and in these unfortunates we find along with the marked functional stigmata of degeneration these actual physical deviations from the normal type which we call anatomical stigmata. But idiocy, insanity, epilepsy, and the like are in themselves conditions which disarrange the nervous co-ordinations so profoundly as to affect the hereditary impulse and give rise to anatomical and functional stigmata in the descendants. What is bequeathed to the degenerate child is a fragile and unstable nervous constitution. The evidence of this inherited fragility of the nervous mechanism may present itself as insanity, or it may be epilepsy, or it may be feeble-mindedness, or it may be criminal tendencies, or it may be simple nervousness, or hysteria, or certain kinds of headaches, or possibly only eccentricity. All of these disorders are more or less interchangeable, and are merely proofs of an unstable nervous organization. Where such conditions do not develop they may still exist in a latent state and pass as a legacy to another generation. Whether the neuropathic state be manifest or latent we are apt to find anatomical stigmata of degeneration present on careful examination.

SIGNIFICANCE OF THE PATHOLOGICAL PALATE AS A STIGMA OF DEGENERATION.

The deformed palate to my mind is one of the chief anatomical stigmata of degeneration. It is true that from this single indication it would not be strictly scientific to adjudge an individual a degenerate. Occasionally, perhaps, a case presents itself where this anatomical stigma alone would suffice to insure a diagnosis of this nature, but usually other stigmata coexist, such as cranial anomalies, deformities of the ear, and the like. The frequency of the pathological palate among marked degenerates, such as insane, idiots, and epileptics, has been testified to by many investigators. Thus Talbot reported forty-three per cent. of abnormal palates in sixteen hundred and five inmates of institutions for the feeble-minded. Ireland makes it nearer fifty per cent. Charon, a later writer than these, found abnormal palates in ten per cent. of apparently normal people, in eighty-two per cent. of idiots and feeble-minded, in seventy-six per cent. of epileptics, in eighty per cent. of cases of insanity in general, in seventy per cent. of the hysterical insane, and in thirty-five per cent. of cases of general paralysis. Nacke has studied particularly the torus palatinus in fourteen hundred and forty-nine individuals, normal and psychopathic. He found it present in 23.9 per cent. of psychopathic women (insane, epileptic, idiot, and criminal); 32.9 per cent. of epileptic women; 34.4 per cent. of criminal women; 22.7 per cent. of normal women.

The percentages were smaller in men than in women. A narrow torus is more common than a broad one.

Stieda examined fifteen hundred skulls for the torus from an anthropological point of view. The skulls were of Prussians, Americans, Africans, Frenchmen, Russians, and Asiatics. He decided that it has no anthropological significance, gives no racial distinction.

While the torus is undoubtedly of value as an index of degeneration, particularly where it is well marked, it probably has less importance in this respect than some of the other forms of pathological palate.

In conclusion, I wish to say that in the anomalies of the palate just described we have not only a physical mark of degeneration, but one of the more common of the anatomical stigmata of this condition. And I would like to suggest here, that if the members of this Society would unite in sending to its museum casts of all the singular palates encountered by them in their professional labors, together with any information they might obtain as to the presence of a highly nervous organization, hysterical tendencies, neurasthenia, epilepsy, feeble-mindedness, idiocy, or insanity, either in the patients themselves or in their immediate relatives, there would be in a few years a priceless collection for some student of this subject to classify, analyze, and draw deductions from, to be presented to your body at some future time.

### LITERATURE.

Morel. Traité des Dégénérescences.

Minot. Embryology.

Talbot. Quoted by Farrar, "Irregularities of the Teeth," New York, 1888.

Charon. Thèse de Paris, July 2, 1891.

Nacke. Arch. f. Psych., vol. xxv., 1890.

Stieda. Internat. Beiträge zur Wissenschaft, Med., Bd. 1.

Calon. Bull. anat. del palato dura Bologna, 1892.

Waldeyer. Ueber den Harten Gaumen, Corr. Blatt der Deutsch Anthrop. Gesellschaft, 1892.

Channing. N. Y. Med. Record, October 3, 1891.

